# Retraction Note: Neuroprotective effect of *Buyang Huanwu Decoction* on spinal ischemia-reperfusion injury in rats is linked with inhibition of cyclin-dependent kinase 5

**DOI:** 10.1186/s12906-022-03750-7

**Published:** 2022-10-13

**Authors:** Lei Wang, Dian-Ming Jiang

**Affiliations:** Department of Orthopedics, First Affiliated Hospital, Chongqing Medical University, #1 Youyi Rd, 400016 Chongqing, China

**Retraction Note: BMC Complement Altern Med 13, 309 (2013)**

10.1186/1472-6882-13-309

The Editor has retracted this article. After publication, concerns were raised regarding blot similarities between different conditions in Fig. 3 C p25 and p35. Further checks by the Publisher have revealed that neither of the authors was affiliated with the institution that issued the Ethics approval. Additionally, the results of the motor function scoring scale reported in Fig. 1 are inconsistent with the description of the method.

The Editor therefore no longer has confidence in the presented data.

Dian-Ming Jiang has not responded to any correspondence from the Editor about this retraction. The Editor has not been able to obtain a current email address for Lei Wang.

